# Associations of Low-Intensity Resistance Training with Body Composition and Lipid Profile in Obese Patients with Type 2 Diabetes

**DOI:** 10.1371/journal.pone.0132959

**Published:** 2015-07-15

**Authors:** Hidetaka Hamasaki, Yu Kawashima, Yoshiki Tamada, Masashi Furuta, Hisayuki Katsuyama, Akahito Sako, Hidekatsu Yanai

**Affiliations:** 1 Department of Internal Medicine, National Center for Global Health and Medicine Kohnodai Hospital, Chiba, Japan; 2 General Internal Medicine, Community Healthcare Studies, Jichi Medical University Graduate School, Tochigi, Japan; 3 Department of Rehabilitation, National Center for Global Health and Medicine Kohnodai Hospital, Chiba, Japan; 4 Department of Nutrition, National Center for Global Health and Medicine Kohnodai Hospital, Chiba, Japan; GDC, GERMANY

## Abstract

Resistance training to increase muscle mass and functional capacity is an integral part of diet and exercise programs for the management of obesity and type 2 diabetes. Low-intensity resistance training with slow movement and tonic force generation (LST) may be a practical and safe regimen for elderly obese individuals but the health benefits are uncertain. This study investigated the effects of LST on body composition and metabolic parameters in obese patients with type 2 diabetes. Twenty-six obese patients with type 2 diabetes engaged in LST training during hospitalization and were advised to maintain this regimen for 12 weeks after discharge. We compared lipid profile, arterial stiffness, and body composition before and after LST training. After 12 weeks of LST training, the ratio of lower extremity muscle mass to body weight increased significantly (0.176 ± 0.028 to 0.184 ± 0.023, mean ± SD), while body fat mass and body fat percentage decreased significantly (36.2 ± 10.9 kg to 34.3 ± 9.4 kg and 41.2 ± 8.6% to 40.1 ± 7.7%, respectively). Moreover, high-density lipoprotein cholesterol was significantly increased (42.2 ± 14 mg/dl to 46.3 ± 12.4 mg/dl) and both free fatty acids and lipoprotein(a) were decreased (665.2 ± 212.1 μEq/l to 525.4 ± 231.3 μEq/l and 15.4 ± 18 mg/dl to 13.8 ± 18 mg/dl, respectively). No significant change was observed in arterial stiffness. Although this study was a non-controlled investigation and some confounding factors including dietary intake, medication and compliance with training might affect the study result, a brief (12-week) LST training program may be a safe and effective strategy for the management of obesity and type 2 diabetes.

## Introduction

Obesity is now a major health problem worldwide. Obese individuals generally have lower muscle strength, which increases the risk of disability [1]. The age-related decrease in muscle mass and accompanying increase in fat mass, termed sarcopenic obesity [2], may also increase the risk of disability and mortality [2,3]. Besides lower muscle strength, obesity is associated with fat infiltration into muscle, contribute to poor lower extremity physical performance [4]. A recent study also showed that lower limb muscle mass was associated with higher visceral fat mass in healthy men [5]. These studies suggest that obesity is perpetuated by poor lower extremity physical performance, and that resistance training (RT) for enhanced lower extremity strength is a good strategy for improving and preventing obesity. Hence resistance training in order to maintain or increase muscle mass and improve physical performance should be an integral part in the management of obesity and metabolic disorders.

Resistance training may also reduce cardiovascular disease (CVD) risk factors such as dyslipidemia and type 2 diabetes [6,7]. The American Diabetes Association recommends that patients with diabetes perform at least 150 min/week of moderate-intensity aerobic exercise as well as RT at least twice per week [8]. Furthermore, a recent meta-analysis concluded that RT improves glycemic control [9]. Thus, RT is recommended for both diabetes prevention and disease management. However, little evidence is available regarding the optimal intensity of RT for obese patients with type 2 diabetes.

A training regimen for gaining muscle size and strength that involved low-intensity resistance exercise with slow movement and tonic force generation (LST) was found to be effective in young men [10]. Previous studies showed that progressive resistance training safely and effectively improved glycemic control, insulin resistance and body composition in patients with type 2 diabetes [11–13]. However, obese patients with type 2 diabetes are recognized as having lower fitness levels than healthy individuals [14], and such patients cannot continue recommended exercise therapy in daily lives. On the other hand, LST training can also be performed without a considerable physical burden and should therefore be beneficial for rehabilitation from orthopedic injuries or as RT among the elderly [10].

To the best of our knowledge, however, no previous studies have examined the changes in body composition and metabolic parameters associated with LST training in obese patients with type 2 diabetes. In this study, we examine the effects of a brief LST training program on body composition and metabolic parameters in obese patients with type 2 diabetes.

## Methods

### Study participants

Of 252 patients with type 2 diabetes admitted to our hospital for glycemic control between September 2013 and August 2014, we recruited 26 eligible patients. We included patients with BMI > 25.0 kg/m^2^, which is defined as obesity by the Japan Society for the Study of Obesity [15], and who agreed to participate in our training program. We excluded patients with physical disability such as osteoarthritis, hyperglycemic crisis, stroke, cardiovascular and respiratory diseases, infectious disease, and malignancy. Patients with edema were excluded because we cannot accurately evaluate body composition using our bioelectrical impedance analysis method. Also excluded were individuals who engaged in regular exercise and/or RT. The Medical Ethics Committee of the National Center for Global Health and Medicine approved the study (Reference No. NCGM-G-001562-01), and all participants provided written informed consent to use their data in the study. The study was performed in accordance with the Declaration of Helsinki.

**Fig 1:**
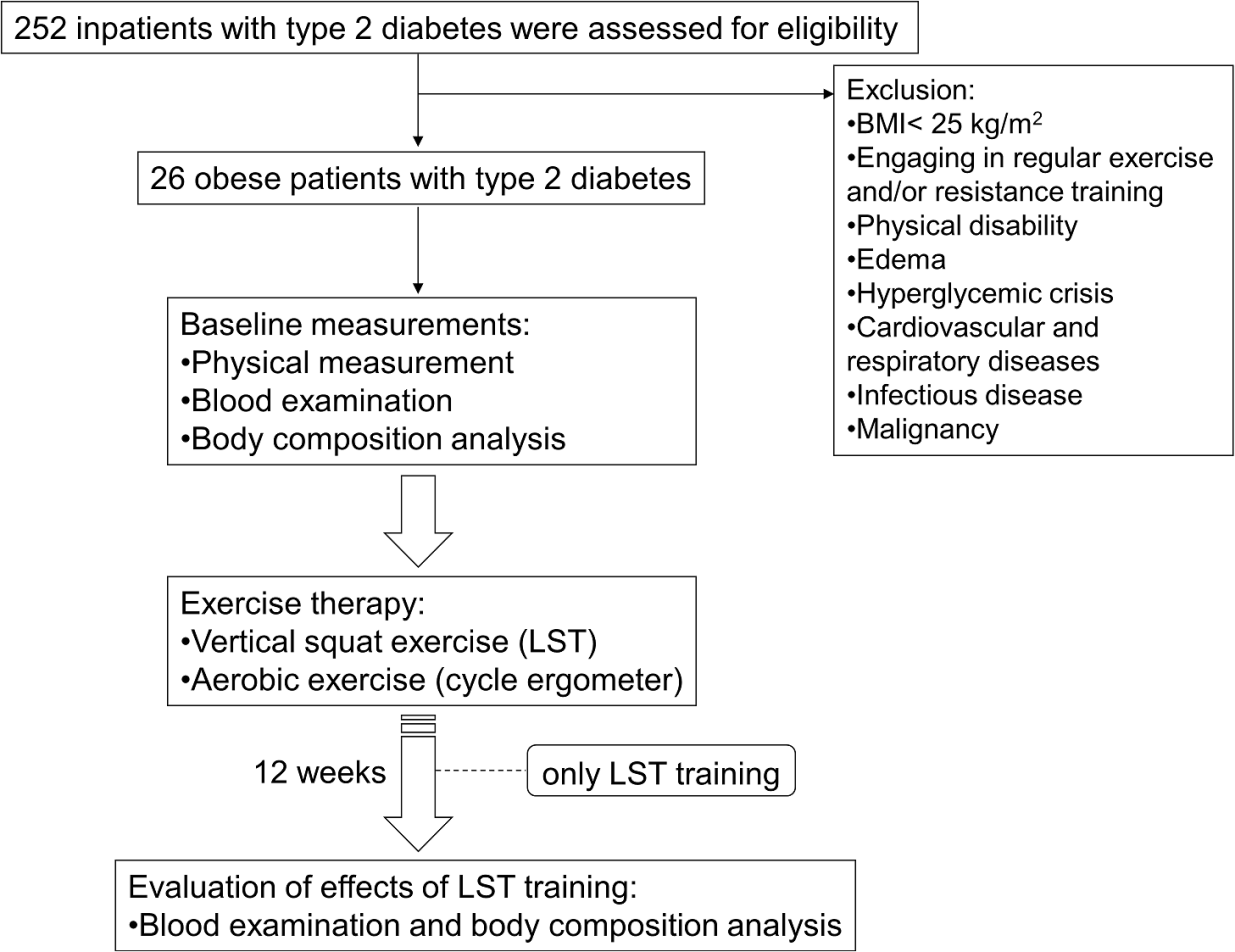
The protocol of this study.

We performed baseline measurements during hospitalization. The participants received standard hospital diets (25−30 kcal/kg) during hospitalization and were instructed by certified nutritional educators to help continue the diet as outpatients. All participants also engaged in RT and aerobic exercise under the instruction of skilled physiotherapists. Approximately 12 weeks after discharge, we evaluated the changes in body composition, anthropometric parameters, and biochemical data. The study protocol is illustrated in Fig 1.

### Training protocol

The participants performed the following vertical half-squat exercise protocol: LST (without free weights) with 3–5 s for eccentric and concentric action, 1 s pause, and no relaxing phase [10]. They performed 8−10 repetitions × 3 sets or as many repetitions as possible. We also prescribed cycle ergometer at 50% of age-predicted maximum heart rate as calculated by the Karvonen method [16] [[(220 - age) - (resting heart rate)] × 50% + resting heart rate] as exercise therapy for diabetes. The average training time was 30–40 minutes per day. They did not engage in any other exercise. All participants performed LST training every other day during hospitalization and were advised to continue LST training only after discharge and were instructed not to take any other form of exercise except LST training. We checked compliance when patients visited our hospital every four weeks and after the 12-week study period.

### Anthropometric measurements

Height and weight were measured with a rigid stadiometer and calibrated scale (seca 764, seca Co., Ltd, Birmingham, United Kingdom). BMI was calculated as body weight (kg) divided by the square of height (m). Waist circumference (WC) was measured in a standing posture at the umbilical level while breathing out.

### Body composition analysis

We analyzed body composition using a bioelectrical impedance analysis device (InBody720, Biospace Co., Ltd, Tokyo, Japan) that measures the relative proportions of lean and fat tissues by differences in tissue impedance. The method is based on the principle that lean body tissue is almost all water and so is a better conductor of electrolytes than fat tissue. Segmental body composition was estimated using a patented 8-point tactile electrode system consisting of platform (feet) and gripping (hand) electrodes. The device uses six frequencies (1, 5, 50, 250, 500, and 1,000 kHz) and produces 30 impedance values for five body segments: right and left upper extremity, trunk, and right and left lower extremity [17].

### Blood examination

Venous blood samples were taken after a 12-h overnight fast. Fasting plasma glucose was measured using an enzymatic method (GA-1170, Arkray, Inc., Kyoto, Japan). Glycated hemoglobin (HbA1c) was measured by high-performance liquid chromatography (HA-8180, Arkray, Inc., Kyoto, Japan). Total cholesterol, free fatty acid (FFA), triglyceride (TG), and high-density lipoprotein-cholesterol (HDL-C) were measured enzymatically using commercially available kits (T-CHO KL for total cholesterol, Sysmex Co., Hyogo, Japan; NEFA-SS for FFA, Eiken Chemical, Tochigi, Japan; Pureauto S TG-N and Cholestest N HDL, respectively, for TG and HDL-C, Sekisui Medical Co., Tokyo, Japan). Low-density lipoprotein-cholesterol (LDL-C) was obtained by the Friedwald formula [18]. Lipoprotein(a) (Lp(a)) was measured by a latex-enhanced turbidimetric immunoassay using Latex Daiichi (Sekisui Medical Co., Ltd, Tokyo, Japan).

### Arterial stiffness and blood pressure

To estimate arterial stiffness, we measured brachial-ankle pulse wave velocity (baPWV) using a blood pressure pulse wave inspection apparatus (BP-203RPEIII, Omron Co., Ltd, Tokyo, Japan). To estimate arterial stiffness and peripheral vascular resistance, we also measured the heart rate-corrected augmentation index (AIx_75_) and central systolic blood pressure (cSBP) using a digital automatic sphygmomanometer (HEM-9000AI, Omron Co., Ltd, Tokyo, Japan).

### Statistical analysis

All statistical analyses were performed using SPSS version 19 (IBM Co., Ltd, Chicago, IL). All values are expressed as mean ± standard deviation (SD). The Wilcoxon signed-rank test was applied to evaluate changes in body composition and other anthropometric and biochemical parameters following the LST regimen. Statistical significance was set at *p* < 0.05.

## Results

### Baseline characteristics

Demographic and baseline clinical characteristics are summarized in Table 1.

**Table 1:** Demographic and baseline clinical characteristics.

**n**	26
**Sex (men / Women)**	11 / 15
**Age (years)**	51.6 years)
**Duration of disease (years)**	6.9 tion
**Length of hospital stay (days)**	20.6 h of
**Height (cm)**	161.9 (cm)
**Weight (kg)**	87.6 t (kg)
**Waist circumference (cm)**	106.5 circum
**BMI (kg/m** ^**2**^ **)**	33.4 kg/mc
**Treatment**	
**Untreated**	5
**Oral hypoglycemic agents**	21
**Insulin**	5
**Anticholesteremic agents**	17
**Antihypertensive agents**	13

Data are expressed as mean ± SD. BMI: body mass index.

No patients dropped out of the training program. The 11 men and 15 women ranged in age from 27 to 75 years and all patients had BMI values > 25 kg/m^2^ (mean 33.4 ± 5.4 kg/m^2^). The majority (16/26, 62%) had HbA1c levels > 8.0%. Most patients (21/26) were currently receiving oral hypoglycemic agents and/or insulin injections, 17 were currently receiving lipid-lowering drugs, and 13 were currently receiving antihypertensive agents. The treatment for dyslipidemia was not changed during the study period. We changed hypoglycemic agents for glycemic control as needed. Daily metformin dosage was increased in 3 patients from 500 mg to 1000 mg, daily insulin glargine dosage was increased in 2 patients (from 15 units to 18 units and from 10 units to 16 units), daily 5 mg of linagliptin was added in one patient, daily 50 mg of miglitol was added in another patient. A patient started insulin therapy with 20 units of insulin glargine and taking daily 2 mg of glimepiride instead of decrease in daily metformin dosage from 1500 mg to 500 mg. In one patient, daily insulin glargine dosage was decreased from 36 units to 20 units. Antihypertensive drugs were changed in 2 patients. One patient started to take angiotensin II receptor blocker, daily 20 mg of olmesartan during the study period. The other patient changed to take an antihypertensive drug from daily 5 mg of amlodipine to daily 20 mg of olmesartan. After discharge, we instructed our patients to take diet directed by nutritional educators during hospitalization, however, some patients could not keep the diet therapy at home.

### Changes in body composition, metabolic risk factors, and arterial stiffness

After 12 weeks of LST training, weight and BMI were significantly lower, although there was no significant change in WC. No significant change in skeletal muscle mass was found, but both body fat mass and body fat percentage decreased significantly, leading to a 4.6% increase in the ratio of lower extremity muscle mass to body weight (Table 2).

**Table 2:** Changes in body composition after LST training.

	Pre-training	Post-training	*p*
**Weight (kg)**	87.6 t (kg)	85.4 t (kg)	0.002
**Waist circumference (cm)**	106.5 circum	103.1 circum	0.184
**BMI (kg/m** ^**2**^ **)**	33.4 kg/mc	32.5 kg/mc	0.002
**Body fat mass (kg)**	36.2 fat ma	34.3 fat m	0.021
**Body fat percentage (%)**	41.2 f 8.6	40.1 f 8.6	0.033
**Upper extremity muscle mass (kg)**	5.81 extre	5.45 extre	0.869
**Lower extremity muscle mass (kg)**	15.5 extr	15.6 extr	0.166
**Ratio of lower extremity muscle mass to body weight**	0.176 of lowe	0.184 of lowe	0.019

Data are expressed as mean ± SD. BMI: body mass index, LST: Low-intensity resistance training with slow movement and tonic force generation.

Plasma HbA1c levels, serum FFA, and Lp(a) were also significantly lower and serum HDL-C significantly was higher after LST training. However, there was no change in peripheral blood pressure, fasting plasma glucose, serum total cholesterol, TG, or LDL-C (Table 3).

**Table 3:** Changes in metabolic parameters.

	Pre-training	Post-training	*p*
**Plasma glucose (mg/dl)**	134.6 gluco	151 6 glu	0.407
**HbA1c (%)**	8.6 c (%)	7.2 c (%)	0.001
**Total cholesterol (mg/dl)**	181.3 choles	182 3 chol	0.378
**Triglycerides (mg/dl)**	153.9 ceride	182.9 ceride	0.284
**HDL cholesterol (mg/dl)**	42.2 hole	46.3 holest	0.002
**LDL cholesterol (mg/dl)**	108.9 oleste	100.5 oleste	0.667
**Free fatty acid (mEq/l)**	665.2 atty ac	525.4 atty ac	0.017
**Lipoprotein(a) (mg/dl)**	15.4 rote	13.8 rote	0.043

Data are expressed as mean ± SD. HDL: high-density lipoprotein, LDL: low-density lipoprotein.

We found no significant change in baPWV (1559 ± 280 cm/s to 1547 ± 292 cm/s, p = 0.484), AIx_75_ (80.1 ± 11.5% to 79.4 ± 14.7%, p = 0.682), or cSBP (135 ± 11 to 130 ± 22 mmHg, p = 0.266).

## Discussion

In this study, a low-intensity resistance training program with slow movement and tonic force generation (LST) increased the ratio of lower extremity muscle mass to body weight, decreased the proportion of body fat, and improved lipid metabolism in obese patients with type 2 diabetes. Although skeletal muscle mass itself did not significantly increase, significant decreases were seen in weight (-2.2 kg), body fat mass (-2.1 kg), and body fat percentage (-1.1%). In sarcopenic obesity, muscle mass decreases while fat mass increases [19]. Thus, to detect sarcopenic obesity, it is necessary to measure not only weight, but also body composition. In addition, individuals with normal body weight but high body fat percentage are more likely to have metabolic syndrome [20] as well as higher CVD risk and all-cause mortality [21], indicating that both decreasing fat mass and increasing lean body mass are important aims in the treatment of metabolic syndrome. Our results also showed that LST training of the lower extremities was effective at maintaining muscle mass in obese patients with type 2 diabetes.

Obesity is associated with increased intramyocellular lipid accumulation in skeletal muscle [22–24] and usually with elevated serum FFA. Resistance training increases intramyocellular lipid oxidation during exercise [25,26], and lower extremity skeletal muscle is a major source of FFA uptake both at rest (15%−20%) and during exercise (30%−60%) [27]. Therefore, RT for enhancing lower extremity muscle mass is effective for improving FFA metabolism. In this study, even in the absence of increased muscle mass, serum FFA levels were significantly lower and HDL-C significantly higher after 12 weeks of LST training. Thus, even low-intensity RT without enhanced muscle mass can improve lipid metabolism in obese patients with type 2 diabetes.

A recent meta-analysis concluded that Lp(a) was an independent and modest risk factor for coronary heart disease and stroke [28]. Results of previous studies investigating the effect of exercise on Lp(a) concentration are controversial [29–34]. Rigla et al. reported a significant decrease in Lp(a) concentration in diabetic patients with higher baseline Lp(a) (> 9 mg/dl) after a 3-month physical exercise program [35], and Muls et al. reported that substantial weight loss in obese women resulted in lower Lp(a) concentration [36]. No previous studies have investigated the effect of low-intensity RT on Lp(a) concentration. To our knowledge, this study is the first to show that LST training also reduces Lp(a) concentrations in obese patients with type 2 diabetes. In the present study, Lp(a) concentrations significantly decreased with weight reduction and with an increased the L/W ratio. Further study is needed to elucidate the potential effects of LST training on Lp(a) concentrations.

High-intensity RT is known to be associated with increased arterial stiffness [37], although the influence of low-intensity RT on arterial stiffness remains unclear. We measured baPWV and AIx_75_ to evaluate arterial stiffness before and after 12 week-LST training. No significant changes were observed in these parameters, which suggests that LST training does not deteriorate arterial stiffness. Although the physiological mechanisms driving the increase in arterial stiffness by resistance training are not known, high-intensity RT stimulates sympathetic nervous system activity and increases arterial blood pressure [37]. LST training is different from high-intensity RT in that participants do not strain during training. It may be because LST training was performed without stimulation of the sympathetic nervous system and elevation of blood pressure that arterial stiffness showed no change. However, this lack of intensity has other benefits. For instance, four older participants (≥ 65 years) were able to perform the exercise program without injury, so this regimen may be suitable for sarcopenic obesity. LST training may have excellent cost-effectiveness because it does not require special instruments and the assistance of other people at home. In present study, the hospitalization for treatment of diabetes including the instruction of LST training cost 132,267 ± 55,554 yen ($1,102 ± 463). Coyle D et al. analyzed the data from the Diabetes Aerobic and Resistance Exercise (DARE) clinical trial and showed that resistance training program cost $38,300 [38]. Although we cannot simply compare the cost-effectiveness of our study with that of the DARE study because the DARE study is a 6 month-randomized controlled trial, the cost-effectiveness of LST training may be high.

This study has several limitations. First, the design of present study does not allow for any specific statement on the effect of LST training on lipid metabolism or body fat distribution because we did not investigate them in control group. Although we instructed our patients to continue the diet therapy after discharge, some patients could not keep the diet therapy, which may have confounded the results. The treatment for dyslipidemia was not changed during the study in all patients. The treatments for diabetes and hypertension were changed as needed, which could also confound our results. Second, we should have assessed insulin sensitivity to understand the associations of low-intensity resistance training with body composition and lipid profile. Third, we did not objectively evaluate patients' physical fitness or their muscle strength using a 1-repitition maximum test; we also did not standardize the training protocol. Forth, we did not supervise the participants engaging in LST training after discharge, so the degree of program compliance may have varied. The training duration was not standardized either. However, patients were instructed sufficiently during hospitalization (85% of the patients performed LST training six times or more) and none of them dropped out, suggesting that they found the routine feasible and that it could be used in the management of obese patients with type 2 diabetes in clinical practice. Since LST training was safe and feasible, we should investigate the effects of LST training in patients with physical disabilities in the future. To clarify the efficacy or non-inferiority to conventional recommended trainings, further studies, preferably randomized controlled trial should be needed in the future.

In conclusion, this study demonstrated that a 12-week LST training program can increase the ratio of lower extremity muscle mass to body weight, decrease body fat, and improve lipid metabolism in obese patients with type 2 diabetes. LST training may be a safe and feasible component of general management for obese patients with type 2 diabetes.

## Supporting Information

S1 File Raw data. (XLS)
