# Correction: Associations of Low-Intensity Resistance Training with Body Composition and Lipid Profile in Obese Patients with Type 2 Diabetes

**DOI:** 10.1371/journal.pone.0137154

**Published:** 2015-08-27

**Authors:** Hidetaka Hamasaki, Yu Kawashima, Yoshiki Tamada, Masashi Furuta, Hisayuki Katsuyama, Akahito Sako, Hidekatsu Yanai

There are errors in Tables 1, 2, and 3. Please see the corrected Tables 1, 2 and 3 here.

**Table 1:** Demographic and baseline clinical characteristics.

n	26
Sex (men / Women)	11/15
Age (years)	51.6 ± 12.5
Duration of disease (years)	6.9 ± 7.0
Length of hospital stay (days)	20.6 ± 9.3
Height (cm)	161.9 ± 8.9
Weight (kg)	87.6 ± 15.7
Waist circumference (cm)	106.5 ± 13.4
BMI (kg/m^2^)	33.4 ± 5.4
Treatment	
Untreated	5
Oral hypoglycemic agents	21
Insulin	5
Anticholesteremic agents	17
Antihypertensive agents	13

Data are expressed as mean ± SD. BMI: body mass index

**Table 2:** Changes in body composition after LST training.

	Pre-training	Post-training	*p*
Weight (kg)	87.6 ± 15.7	85.4 ± 15.1	0.002
Waist circumference (cm)	106.5 ± 13.4	103.1 ± 12.8	0.184
BMI (kg/m^2^)	33.4 ± 5.4	32.5 ± 4.8	0.002
Body fat mass (kg)	36.2 ± 10.9	34.3 ± 9.4	0.021
Body fat percentage (%)	41.2 ± 8.6	40.1 ± 7.7	0.033
Upper extremity muscle mass (kg)	5.81 ± 1.28	5.45 ± 1.71	0.869
Lower extremity muscle mass (kg)	15.5 ± 3.0	15.6 ± 3.3	0.166
Ratio of lower extremity muscle mass to body weight	0.176 ± 0.028	0.184 ± 0.023	0.019

Data are expressed as mean ± SD. BMI: body mass index

LST: Low-intensity resistance training with slow movement and tonic force generation

**Table 3:** Changes in metabolic parameters.

	Pre-training	Post-training	*p*
Plasma glucose (mg/dl)	134.6 ± 47.2	151 ± 63.4	0.407
HbA1c (%)	8.6 ± 2.4	7.2 ± 1.2	0.001
Total cholesterol (mg/dl)	181.3 ± 42.2	182 ± 38.1	0.378
Triglycerides (mg/dl)	153.9 ± 57.8	182.9 ± 96.6	0.284
HDL cholesterol (mg/dl)	42.2 ± 14	46.3 ± 12.4	0.002
LDL cholesterol (mg/dl)	108.9 ± 32.6	100.5 ± 24.2	0.667
Free fatty acid (μEq/l)	665.2 ± 212.1	525.4 ± 231.3	0.017
Lipoprotein(a) (mg/dl)	15.4 ± 18	13.8 ± 18	0.043

Data are expressed as mean ± SD. HDL: high-density lipoprotein, LDL: low-density lipoprotein
